# A Patient‐Centric, Digital Multimodal Post‐Market Approval Continuous Monitoring of Drug Safety & Efficacy Platform for Blarcamesine

**DOI:** 10.1002/alz70859_106459

**Published:** 2025-12-26

**Authors:** Audrey Gabelle, Marwan N. Sabbagh, Timo Grimmer, Luca Villa, Elizabeth Gordon, Mathilde Borrot, Ayoub Gueddou, Nicolas Guizard, Olivier Courreges, Kun Jin, William R. Chezem, Juan Carlos Lopez‐Talavera, Christopher U Missling

**Affiliations:** ^1^ Montpellier Excellence University, Montpellier France; ^2^ Barrow Neurological Institute, Phoenix, AZ USA; ^3^ Technical University of Munich, School of Medicine and Health, TUM University Hospital, Center for Cognitive Disorders, Munich, Bavaria Germany; ^4^ Qynapse, Paris, Île‐de‐France France; ^5^ Anavex Life Sciences Corp., New York, NY USA

## Abstract

**Background:**

There is a critical need to establish robust Real‐World Evidence (RWE) platform for monitoring both clinically relevant benefits and potential mid/long‐term side effects of Alzheimer’s disease (AD) drugs at an individual level, with the ambition to enhance personalized medicine in AD. However, there are no standards for digital post‐market approval continuous monitoring of drug safety and efficacy. Oral blarcamesine, a novel convenient scalable precision medicine treatment was submitted and accepted for Marketing Authorisation Application (MAA) review by the European Medicines Agency. To innovate the monitoring of blarcamesine, we built a patient‐centric, digital multimodal platform based on three pillars consisting of digital Patient Reported Outcomes (PRO), digital clinical and cognitive assessments, and automated MRI Brain Imaging exams.

**Method:**

The digital PRO includes regularly administered surveys via wearable device data to provide a comprehensive view of treatment impact. The digital clinical and cognitive assessment integrates interactive applications for clinical and cognitive training and performance tracking and historical comparisons. The brain imaging pillar implements automated MRI image quality control and automated monitoring of brain volume changes using the QyScore ® neuroimaging analysis platform from Qynapse.

**Result:**

Such a platform would enable continuous, real‐time data collection from patients, facilitating more precise tracking of drug efficacy, patient individual responses, and the emergence of potential side effects.

**Conclusion:**

This post‐market approval continuous monitoring platform may benefit all stakeholders, including patients, clinicians, regulators, service providers and payors. This modularly digital platform can be customized to fit also other post‐market approval monitoring.

